# The current state, opportunities and challenges for upscaling private investment in biodiversity in Europe

**DOI:** 10.1038/s41559-024-02632-0

**Published:** 2025-02-05

**Authors:** Sophus O.S.E. zu Ermgassen, Isobel Hawkins, Thomas Lundhede, Qian Liu, Bo Jellesmark Thorsen, Joseph W. Bull

**Affiliations:** 1 Nature-positive Hub, Department of Biology, University of Oxford, Oxford, UK; 2 Leverhulme Centre for Nature Recovery, University of Oxford, Oxford, UK; 3 Department of Food and Resource Economics, University of Copenhagen, Copenhagen, Denmark

**Keywords:** Sustainability, Conservation biology

## Abstract

European countries have committed to ambitious upscaling of privately funded nature conservation. We review the status and drivers of biodiversity finance in Europe. By implementing semistructured interviews with 25 biodiversity finance key informants and three focus groups across Europe, we explore opportunities and challenges for upscaling private investment in nature. Opportunities arise from macroeconomic and regulatory changes, along with various technological and financial innovations and growing professional experience. However, persistent barriers to upscaling include the ongoing lack of highly profitable investment opportunities and the multitude of risks facing investors, including political, ecological and reputational risks influencing supply and demand of investment opportunities. Public policy plays the foundational role in creating and hindering these mechanisms. Public policy can create nature markets and investment opportunities, meanwhile agricultural subsidies and poor coordination between public funding sources undermine the supply of return-seeking investment opportunities. Investors demand derisking investments from uncertainties; in part caused by political uncertainty. These markets require profound state intervention to enable upscaling whilst achieving positive ecological outcomes; private investment will probably not upscale without major public policy change and public investment.

## Abstract (web summary)

Semistructured interviews and focus groups with experts in biodiversity finance in Europe identify opportunities and barriers to upscaling private investment in biodiversity, and emphasize that public policy is the key to enabling progression.

## Main

Biodiversity globally continues its trajectory of long-term decline^1^. One of the many transformative changes required to reverse this trend and achieve global conservation goals is to address vast mismatches between the level of investment in nature conservation and restoration, and current spending on conservation and perverse government subsidies that incentivize further declines^2–4^. Addressing these economic drivers and enablers of biodiversity loss and recovery is central to the Kunming-Montreal Agreement, with targets 14, 15, 16, 18 and 19 all targeting harmful subsidies, the need of businesses and financial institutions to assess and address their impacts and dependencies on nature and upscaling investment. Public funding for conservation is not growing at close to the rates required to address biodiversity loss^5,6^. Therefore, in the Kunming-Montreal agreement and prevailing policy discourse^7–12^, there is a strong emphasis on upscaling private investment in conservation and restoration, through “Leveraging private finance, promoting blended finance … green bonds, biodiversity offsets and credits (Target 19)”. However, mechanisms for attracting private investment into nature conservation and restoration remain controversial and understudied^12–14^.

Nowhere is this more apparent than in Europe (including the United Kingdom), perceived as a global leader in sustainability policy^15^, put into operation through ambitious policies to address biodiversity declines such as the Green Deal, EU Forest Strategy and EU Nature Restoration Law. However, there is little research into private investment in nature in Europe^16^. A recent global review of empirical studies into incentive mechanisms for forest restoration found zero studies from Europe^17^ and the Biodiversity Finance Initiative of the United Nations Development Programme, a leading database on biodiversity finance instruments globally, contains no schemes from Europe^18^. Previous work on investors’ attitudes has focused largely on exploring their motivations for engagement and on investments from institutions in the global north into the global south, with little on investments domestically or within Europe^19^. Here we address that gap by first reviewing the current state of biodiversity finance in Europe, then presenting the results of three focus group discussions and 25 semistructured interviews with biodiversity finance experts (23 in leadership roles in their organizations) across Europe investigating the barriers and opportunities for upscaling private investment in nature.

## Overview of biodiversity finance in Europe

Europe (alongside Latin America) is currently the world region that spends least on biodiversity protection as a percentage of its national budgets globally (an estimated 0.1%)^2^. Analysing budget data, the European Commission estimates that a total €144 billion was spent on biodiversity in the European Union (EU) from 2014 to 2019 (ref. ^20^), although this assessment used an investment tagging approach which is sensitive to subjective classifications^21^. It includes €70.4 billion from the European Commission for domestic initiatives (mostly via the Common Agricultural Policy which is recognized to have done little to address biodiversity loss^22^) and €2.2 billion for international projects aimed at global environmental goals. Member states contributed €59.0 billion domestically and €12.9 billion internationally. Extrapolating from these figures, the average annual spending on biodiversity from 2021 to 2030 is estimated at €29.4 billion. However, the EU Biodiversity Strategy for 2030 indicates a projected annual requirement of €48.15 billion, implying an annual shortfall of €18.7 billion from 2021 to 2030. In the United Kingdom, the public sector spends approximately £700 million per year on conservation (0.031% of gross domestic product, down from 0.037% in 2008/9)^6,23^, with a recent industry-led assessment suggesting a biodiversity finance shortfall of approximately £5.6 billion per year^24^.

Biodiversity is typically conceptualized as a public good, subject to market failures (or ‘cost-shifting’ in ecological economics^25^) because its value for society is not captured in conventional markets and is therefore not effectively internalized into the prices of goods and services^26^. However, biodiversity underpins the ecosystem services upon which the economy depends, and therefore the loss of biodiversity represents a risk to economic activity^27,28^. This underlies efforts to persuade businesses and the financial sector that nature loss represents a material risk, and is therefore worthy of private investment. Historically dominant motivations for private investment in nature have been investing to address risks (including supply chain risks, regulatory risks and reputational risks)^29^, for corporate social responsibility, marketing purposes or investing in carbon offsets for organizational net-zero strategies^19^. However, incentives facing organizations and investors are changing as a result of a rapidly evolving regulatory landscape and emerging voluntary initiatives at global, European and national scales (Table 1).

**Table 1:** A sample of key legislative and voluntary drivers of potential increases in private investment in nature conservation/restoration in Europe

Scope	Policy or initiative	Target of initiative	Driver of private investment
Global	Kunming-Montreal post-2020 global biodiversity framework	All national signatories of the UN Convention on Biological Diversity	Target 19 explicitly calls for “Significantly increasing domestic resource mobilization, facilitated by the preparation and implementation of national biodiversity finance plans or similar instruments according to national needs, priorities and circumstances …. Leveraging private finance, promoting blended finance, implementing strategies for raising new and additional resources, and encouraging the private sector to invest in biodiversity, including through impact funds and other instruments …. Stimulating innovative schemes such as payment for ecosystem services, green bonds, biodiversity offsets and credits …”. Targets 14, 15 and 18 all also mention increasing positive impacts, scaling up incentives and aligning fiscal flows with overall biodiversity goals.
Global, voluntary	Taskforce for nature-related financial disclosure (TNFD)	Businesses	Organizations encouraged to report on their nature-related opportunities. These include “Amount of capital expenditure, financing or investment deployed towards nature-related opportunities, by type of opportunity, with reference to a government or regulator green investment taxonomy or third-party industry or NGO taxonomy, where relevant …. Increase and proportion of revenue from products and services producing demonstrable positive impacts on nature with a description of impacts”. Core metrics for organizations reporting information aligned with the TNFD include their total spatial footprint, including their ‘total rehabilitated/restored area’ and their change in extent, as well as indicators of the state of nature within which the company operates (using a flexible set of contextually appropriate indicators).
EU	EU biodiversity strategy for 2030	EU member states	Section 3.3.2. highlights the desire to upscale private investment. Highlights that at least €10 billion will be mobilized through blended finance via the InvestEU initiative. Promotes the role of the EU taxonomy aiming to provide “long-term certainty for investors and help embed sustainability in the financial system”. Advocates for changes to tax and pricing systems to reflect ‘user pays’ and ‘polluter pays’, and advocates for the use of state procurement to drive demand for companies and products that deliver nature-based solutions.
EU	Nature restoration law	EU member states	Member states will be asked to include estimates of financing needs and the means of intended financing, including private finance, in their national restoration plans and report on the implementation every 3 years.
EU	EU forest strategy for 2030	EU member states	Section 3.4. covers financial incentives for forest owners to improve the quantity and quality of EU forests. Strategy advocates for increasing subsidies for more ecological forms of forest management, as well as upscaling carbon farming initiatives “through the generation of carbon certificates that can be traded in markets”. Highlight that the EU Commission is developing a regulatory framework for certifying carbon removals.
EU	Corporate sustainability reporting directive (CSRD)	Large businesses	Disclosure requirement E4-1. Organizations will be asked to disclose a description of the resilience of their strategy and business model in relation to biodiversity and ecosystems. This includes potentially disclosing a transition plan demonstrating how their business model will be adjusted to be compatible with the EU biodiversity strategy or the Kunming-Montreal Agreement. Requirement E4-3 requires they disclose their biodiversity and ecosystems-related actions, including resources devoted to these actions.
EU	Carbon removals and carbon farming certification (CRCF) regulation	EU member states	Regulation aiming to “improve the EU’s capacity to quantify, monitor and verify the authenticity of … carbon removals. In particular, it sets out rules to recognize certification schemes …”. The Commission highlights that “certified carbon removals can be the basis of new economic opportunities, and can be monetized through private schemes and public sector support, as well as generating commercial advantages with consumers looking to reward environmentally-friendly practices. Carbon farming will create new business models for farmers and foresters and is expected to yield significant benefits for biodiversity”.
England (nature policy in the United Kingdom is devolved)	Nature markets framework	Government strategy	Outlines the government’s plans to scale-up private investment in conservation and restoration and construct ‘high-integrity’ market-like mechanisms.
England	Biodiversity net gain	Domestic construction industry	Most new developments in England need to demonstrate that they will leave biodiversity at least 10% better off after development than beforehand, as measured using the government’s ‘statutory biodiversity metric’. If they cannot meet this obligation within the boundaries of their development, they will need to purchase biodiversity units from a new national biodiversity market.

A precondition for attracting return-seeking private investment into nature conservation is that the conservation or land management activities delivered through that investment must generate cashflows or prevent costs^30,31^. Financing instruments and strategies for investing in biodiversity-related outcomes are proliferating rapidly^3,7,30–34^. These include the growing number of nature-related funds, which are predominantly focused on the generation of market goods in theory associated with biodiversity cobenefits (that is, agriculture or forestry)^35^, green bonds^36^ and emerging mechanisms such as biodiversity credits^37^. However, ensuring that these private investment strategies are in reality delivering improvements in biodiversity remains extremely challenging^38^. For example, funds invested in sustainability-certified agriculture or forestry, commonly rely on certification for their biodiversity impacts, despite limited evidence for effectiveness^39,40^. For green bonds, work analysing how these actually generate revenues has found that often the link between their activities and real-world biodiversity outcomes is tenuous^38,41^.

**Fig. 1:**
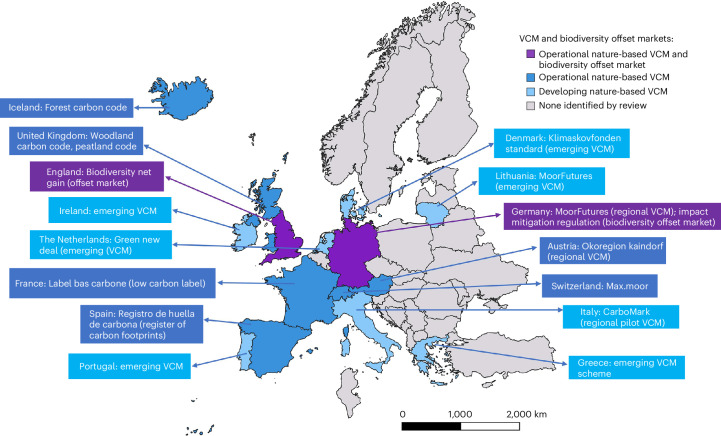
Overview of state-affiliated domestic carbon and biodiversity offset markets in Europe. Markets are those for which information was available in English (Methods). Several national biodiversity offsetting markets are also in development.

In contrast with nature-focused investments, which aim to deliver biodiversity cobenefits most commonly as a biproduct of producing market goods, the last few decades have witnessed a rapid proliferation of instruments for commodifying direct increases in biodiversity or carbon (or both) to create potential revenue streams from delivering improvements in nature. These include the expansion of biodiversity and carbon offsetting market-like mechanisms^42,43^, perceived as the simplest classes of financial instruments for conservation to upscale^3^ and are therefore core to the ambitions for conservation funding of many countries^8^. Although the ecological benefits of these market mechanisms are generally variable^44,45^, no counterfactual-based evaluations to date have been conducted in Europe^16^. A suite of countries in Europe now have domestic carbon or biodiversity markets underpinned by the state, with several others encouraging voluntary purchase of biodiversity offsets which is often a precursor to these becoming embedded in legislation (Fig. 1).

## Results and discussion

From our interviews with 25 biodiversity finance experts across Europe we identified two key themes regarding the opportunities for upscaling private investment: (1) macroeconomic and political factors driving potential increases in both supply and demand of projects for private finance and (2) various sources of innovation and specialized experience that emerge as these markets develop.

### Major economic shifts driving private investment

In our sample, the intensification of rhetoric and efforts to upscale private investment in conservation were fundamentally perceived to be driven by increasing public awareness and demand for policies addressing biodiversity loss, coupled with a lack of trust that governments possessed the capacity or political capital required to directly address the problem through increasing public spending on biodiversity conservation. “So we can continue to think and expect that governments will solve our problems, but they don’t. They have consistently proven in the last 50 years of my existence here on Earth, they don’t … so you need to focus on private markets, and you need to make that work” (P2, nature market broker).

Our interviews pointed to a range of potential drivers of incentives for increased demand for investments in conservation. These included the traditional motivations of corporate social responsibility and marketing^19^, organizational net-zero and nature-positive commitments^46^, as well as the voluntary and policy drivers outlined above such as the taskforce for nature-related financial disclosure (TNFD) and CSRD. We noted that interviewees did not substantiate the exact mechanisms through which they thought these supportive policies and initiatives were to drive demand for conservation investment^47^. Interviewees highlighted interactions between these different mechanisms, with voluntary corporate initiatives often seen as a necessary precursor to the adoption of supporting policies in legislation. Risk management was another key driver^29,48^, with dependencies of companies on nature especially in corporate supply chains perceived as an increasingly powerful lever for making biodiversity loss material to companies and therefore drive demand for nature-related investments. An increasing number of commodification mechanisms and policies were seen to be creating opportunities to derive cashflows from biodiversity improvements (for example, new and increasingly established nature markets such as biodiversity net gain in England), increasing investability.

On the supply side, interviewees noted the increasing regulation and economic pressures on farming, driving a perceived interest from land managers to diversify their income streams and participate in environmental markets. Additionally, interviewees and focus group participants noted interactions between consolidated land ownership and participation in environmental markets, with landholders or organizations with large non-operational landholdings looking to use these markets as opportunities to generate revenues through land ownership.

### Opportunities arising from innovation and experience

Alongside large-scale ‘push–pull’ factors, various innovations and the development of experience within the biodiversity finance sector were perceived as key enablers of further upscaling. Technological innovation including advancements in near real-time biodiversity monitoring (interviewees mentioned images taken through smartphones and standardized sampling protocols, bioacoustics, eDNA and remote sensing) were seen as central^49^. Interviewees believed it would enable demand by increasing buyers’ ability to obtain evidence that their contributions to biodiversity improvements mattered. However, although several project developers and brokers described in detail their technologically enabled data collection strategies, these interviewees did not describe how counterfactuals would be ensured for valid impact detection. Less attention was paid in our sample to additionality than to monitoring.

Other non-technological forms of innovation were also found to be critical. Interviewees alluded to new markets and asset classes taking a long time to develop and reach maturity, and there was a sense of ‘learning by doing’, leading to the generation of new financial innovations and logistical or legal innovations to enable these new investment classes. These included fund aggregation^9^ and new types of governance structures such as innovative contract designs including ecological conditionalities and insurance to attempt to guarantee the delivery of the biodiversity enhancement underpinning the investments^50^. Another key element was the development of skilled practitioners with combinations of conservation and finance expertise to broker between the two disciplines^51^.

The analysis of interviews demonstrate opportunities for upscaling private investment, especially in the context of increasing economic pressures on farming driving increased interest in diversification and rapid industry innovation. However, our interview analysis indicates that key links are still missing between these conceptual opportunities and real investments delivering scientifically credible results. For example, there is a lack of clarity on the tangible relationships between hypothetical demand drivers such as disclosure frameworks and how exactly these would drive increased investment or on how ecological monitoring enables the estimation of impact.

### Challenges to upscaling high-integrity investment

Upscaling private investment in nature faces myriad challenges. In our interviews the overarching themes were the lack of highly profitable investment opportunities and inherent mismatches between the perceived realities of risks and uncertainties of doing conservation in practice and the level of risk that private investors are willing to bear. Conservation is subject to many categories of risk^38^ and for investments in conservation to deliver risk-adjusted returns competitive with other options available to investors, the cashflows from private investments need to be either sufficiently high to justify the risks^31,33^ or the risks need to be mitigated (for example, derisking via blended finance). Otherwise, as one interviewee stated: “they [financial institutions] have easier ways of making money” (P20, team leader at a multilateral development institution). One recent paper demonstrated for a biodiversity-focused private equity firm, investments which were nominally delivering biodiversity improvements had a mean target internal rate of return of 14.7% and blended finance deals subject to derisking had a mean of 11.9% (ref. ^33^). This corroborated evidence from our interviews highlighting the very high returns required to justify the risks of investment and a fundamental lack of mechanisms that can deliver sufficiently high revenues. “I would consider us very risk-tolerant investors, very, in the grand scheme of things. And we’re looking at things that others would definitely not look at. But even for us, so many of the projects we see are just … It’s not clear where the revenues are going to come from” (P17, investor).

We identified three subthemes relating to risk in our interviews: risks relating to politics and regulation, risks stemming from the mismatch between the complexity of ecology and the needs of investors, and risks stemming from social perceptions and inequities.

#### Political and regulatory risk

Interviewees noted that the ecological success of mechanisms for attracting private investment into conservation would require political will and substantial investment in ensuring high-quality governance, just as public-sector-led conservation investment would^52^; except one interviewee (P2, nature market broker) who felt state intervention would undermine their effectiveness. Nearly all participants noted therefore that the development of market-based approaches for conservation financing are a complement, not a substitute for genuine political will for addressing biodiversity loss; the ecological outcomes of either state-led or private-led investments in conservation outcomes hinge on that same political will^31,52^. Inconsistent political will to address biodiversity loss translates into widespread regulatory uncertainty, a key barrier to both supply and demand for conservation outcomes and therefore to the certainty of cashflows generated through such investments. On the demand side, investors seek a high degree of confidence that there will be a market for the biodiversity benefits generated so that they can sell commodified biodiversity increases to generate cashflows, but the looming threat of governments weakening or removing legislation that is the driver of this demand was a major driver of risk. On the supply side, this same regulatory uncertainty was perceived as a large barrier to initiating the enrolment of land managers into delivering conservation land management.

#### Mismatches between finance and ecology

Some conventional critiques around the commodification of nature appeared in our interview dataset of interviews (for example, relating to non-fungibility, unsuitability for some types of biodiversity, emphasis on carbon over biodiversity and risks of greenwashing)^53–59^. Beyond these concerns, there were additional risks hindering the upscaling of investment. A main barrier to investment cited by investors was the cost of monitoring and the lack of ecologically realistic metrics to evidence increases in biodiversity^51^. Therefore, investors often use proxies for signalling the biodiversity value of the investment, which might be some form of sustainability certification in the case of agriculture or forestry-related funds^35^ or biodiversity or carbon metrics aligned with offset certification schemes or national legislation. However, periodic impact evaluations demonstrate that many of the proxies on which they rely often overstate the contributions of these investments to enhancing nature^16,39,40,55,60,61^. The lack of accepted metrics is a barrier, as interviewees noted, because even well-intentioned purchasing of biodiversity or carbon outcomes using a commodification mechanism or key performance indicators, may present a reputational risk if subjected to public criticism. Participants and focus groups highlighted that carbon credits were by far the main well-developed bankable revenue streams to date, presenting challenges as the kinds of projects and ecologies optimizing for carbon were misaligned with those optimizing for biodiversity.

Additionally, interviewees noted both spatial and temporal mismatches between conservation and the needs of investors. Temporally, conservation was perceived as requiring large up-front investments for uncertain long-term payoffs^11^, a challenge both because of uncertainties regarding the long-term potential revenues from the investment, and because enroling in selling biodiversity-related outcomes meant land managers would have to forgo their existing revenue streams in the short run, reducing their incentives to enrol. One interviewee noted that this alone meant such projects were probably better matched to receiving non-commercial, long-term public investment. Spatially, interviewees noted that areas of high biodiversity value tend to be located in areas of low human pressure and weak institutions, highlighting that these are the very places where institutions are probably too weak to give investors confidence^62^ (in the context of European investments in the global south).

#### Risks from social perceptions and inequities

Interviewees and focus groups noted that those best positioned to take advantage of the opportunities created by upscaling private investment opportunities were institutions with rights over key limiting factors, such as land, or previous experience of social and environmental management for large land-based projects, such as businesses with large non-operational estates; therefore interviewees acknowledged that expanding biodiversity-related investment opportunities have the potential to exacerbate pre-existing inequities. Engagement with local communities at project sites was consistently mentioned by project developers as something that was a ‘nice-to-have’, but resource shortages frequently meant that these activities were deprioritized beyond just satisfying the basic requirements of legislation or accreditation schemes. On the other hand an investment advisor (P5) argued that effective social engagement was an essential risk management tool, as they perceived effective management of social risks to correlate with the good governance required to address other project risks. Additionally, interviewees recognized that inequities may pose a reputational risk to the credibility of these markets themselves through public opinion, especially in the context of blended finance, in cases where public funding backed by taxpayers would be used to derisk investments for financial institutions^63^.

### The role of public policy

The role of public policy was emphasized by all interviewees. A diversity of views were represented, from perceptions of government as the stifler of market innovation and real action to address biodiversity loss (P2, nature markets broker), to government’s emphasis on scaling up private investment being a ‘symbolic instrument’^42^ designed to impose minimal disruption and ultimately legitimize prevailing unsustainable business practices (P19, director of a sustainable finance non-governmental organization (NGO)), through to cautious optimism about the attempts of public policy to internalize biodiversity into business and investment decisions contingent on high-quality governance and enforcement (most participants across all stakeholder groups). We identified two core themes: recognizing biodiversity finance as just one small part of the conservation puzzle and identifying that public policy is the key enabler of biodiversity finance. Public policy is presented as the creator of these private investment opportunities yet also their major threat.

#### Upscaling private finance no substitute for regulation

Interviewees from across all stakeholder groups frequently caveated their expectations around upscaling biodiversity finance by situating it as just one piece of the public policy landscape required to address biodiversity loss. They reiterated the public good nature of biodiversity and the challenge in commodifying most types of biodiversity, and therefore highlighted that public investment remained essential. They recognized opportunities for improving the effectiveness of public investment, including through results-based budgeting. Interviewees emphasized the importance of governments strengthening regulations to prevent biodiversity loss, even arguing that this would help create more opportunities for investors as profitable innovation would be required to overcome constraints created through direct regulation of ecological harms^64,65^: “So there’s a set of things that should just be banned in my view, and that will really help because it’ll help investors. It will drive up demand for, for example, products that can revitalize highly degraded soil. Why is there less demand for that? Because there is still an option of additional conversion across the world” (P17, investor). This investor-proposed perspective is notable for highlighting that regulation can be a source of innovation rather than merely an economic constraint^64,65^.

Interviews identified many policies that could be enacted to both address biodiversity loss and facilitate the development of private investment opportunities, including improved data transparency on land management activities^66^, changes in taxation regimes, financial regulation and supervision^67^ and investment in biodiversity-related state capacity and skills^68^ (Supplementary Note).

#### Public policy as enabler of upscaling and its own worst enemy

Public policy was perceived as the dominant driver of opportunities for investing in nature. Through the creation of biodiversity-related markets, public institutions were framed as the mediators of the outcomes of private finance through the design of well-designed commodification mechanisms that would be aligned with delivering positive outcomes coupled with effective enforcement. Additionally, public funding was seen as necessary to catalyse market opportunities through direct subsidies for projects attempting to enter nascent nature markets and derisking investments in these projects. These perspectives challenge the view that there is a dichotomy between public and private biodiversity finance—substantial public investment in creating, governing and stimulating demand for the markets that public policy itself created was perceived as fundamentally essential: “That is [a change] you’ve got to instigate that at a system level. I don’t think again, it’s one that’s going to happen organically within our economy on its own. Our economy, every economic actor plays according to the existing rules of the game. And yes, some innovation happens that changes that and pushes people on and makes things move. But, ultimately they’re constrained by the operating environment within which they are, within which they exist … And, I think the only force that can fundamentally shift that reality is through the role of the state” (P16, team lead at international eNGO).

Given the overwhelming role of public policy, effective governance and well-coordinated regulations were viewed as critical. However, interviewees and focus groups highlighted fundamental tensions between different public policies that appear to both stimulate private investment opportunities and suppress them. One key tension related to the need for access to land to implement projects for nature markets and public policy as a major barrier to land acquisition or enrolment. Nature investment requires the enrolment of land, yet in some jurisdictions restrictions on using public money to purchase land meant that nature investment projects that included land acquisition as part of their conservation management were unable to access public funding or support, preventing projects from proceeding^9^. Additionally, agricultural subsidies were perceived as a major barrier to enroling land in conservation management. In providing land managers with stable public-policy-derived incomes, enrolment in nature projects must deliver a business case that exceeds the opportunity cost, which spans not just the revenues from subsidies but also the long-term stability of those payments. Subsidies were perceived to be internalized into land prices, increasing the value of land, reducing the viability of privately funded conservation management. On one hand, public policy aims to create and scale nature markets; on the other, through the subsidy system governments invest in its main competition for land: “… the people who are really going to be making money from this are the ones with the most limiting factor in the whole system, which is land” (P24, team lead at conservation charity).

A second key tension identified was between the derisking being demanded from governments to help address the uncertainties facing investors and governments being one of the key causes of these uncertainties. Political and regulatory uncertainty increases risk for investors, as does the uncertainty of how well these markets will be enforced in practice and therefore whether investors will end up investing in something that is later exposed as being non-compliant, highlighting the importance of consistent and long-term policy signals and regulatory certainty for upscaling markets.

Last, a critical subtheme was poor coordination between different sources of finance (that is, non-return-seeking grants and subsidies and private finance opportunities) that leads to competition between existing sources of funding, with projects having a preference for non-return-seeking grants^9^. A lack of coordination can lead to projects, which were strong candidates for return-seeking finance ending up funded by direct grants, drawing potential supply out of markets. This highlights the need for better coordination at landscape scales between funding sources to direct different kinds of financing into different projects based on the ecological characteristics of the projects and their suitability for funding through existing market-based funding mechanisms.

Our study has generated new insights and reinforced existing ones, presenting a snapshot of the drivers and policies underpinning private investment in biodiversity in Europe. The private investment ecosystem is evolving and maturing, underpinned by perceived macroeconomic opportunities that prompt land managers to investigate diversifying into generating biodiversity-related commodities. However, we still identify missing links between hypothesized demand drivers and real investments in biodiversity, such as incomplete conceptualizations of how biodiversity disclosure might cause increased private investment, a severe lack of opportunities to derive cashflows from biodiversity and myriad risks including the under-explored risk arising from the perception that biodiversity markets may exacerbate inequities and privilege those owning land.

This study reinforces that public policy is the critical enabler of private investment, through creating the commodification mechanisms that underpin markets, regulation to prevent enrolment of poor projects and then even stimulating demand through derisking and blended finance to bring nature investment projects up to the very high-risk-adjusted returns required to attract mainstream private investment. Therefore, although these markets have arisen as a ‘second-best’ solution to better direct public regulation and investment in biodiversity-related public goods, their success remains contingent on similar political will and substantial public investment. It remains an open question whether solving the nature finance gap through the expansion of public policy-derived private investment opportunities or direct public policy and public investment is the more cost-effective solution to achieving biodiversity funding goals^69^.

This work highlights many avenues for future research, including empirical explorations of the interactions between agricultural subsidies and nature markets, the appropriate role of derisking in nature markets to maximize value to society and how to coordinate across different funding streams to direct the right kinds of capital into the right places to deliver on overarching biodiversity goals.

## Methods

### Review of national biodiversity and carbon offset policies

No comprehensive up-to-date review exists for national voluntary carbon markets (VCM) and biodiversity offset markets in Europe. Consequently, to obtain information on national policies, it was necessary to use a variety of data sources identified through a search of scientific and grey literature using Google and Google Scholar. A key limitation of these methods is that only sources in English and translated webpages were used.

#### National VCMs

To investigate domestic nature-based VCMs in Europe, we conducted a literature review. We searched the Carbon Gap Interactive Policy Tracker and World Bank Carbon Pricing^70^ reports supplemented by an online search of both scientific and grey literature on each European country, using keywords and phrases: ‘VCM’, ‘voluntary carbon market’, ‘national carbon market’, ‘domestic carbon market’, ‘carbon offset market’, ‘voluntary carbon offsets’ and ‘national carbon offsets’. For each country, a search was conducted first in English. Following this, the same searches were repeated in the national language(s) of each country, using Google Translate to translate the key phrases ‘carbon offset market’ and ‘biodiversity offset market’. We searched relevant sources identified for reference to a domestic-state-supported VCM involving the sale of carbon credits delivered through nature-based climate solutions. VCMs are included where credits are delivered through restoration of any type of natural ecosystem. Where a domestic VCM was identified, we determined where possible: whether the market operates at a national or subnational level; whether the market is in an operational or developmental stage; the ecosystem type(s) through which carbon credits are delivered. Market-based mechanisms operating at an international level or those involving sale of non-nature-based credits in a compliance emissions trading scheme (ETS), are not included in this review.

#### Compliance emissions trading schemes

In addition to national VCMs, countries may sell carbon credits on the international voluntary carbon market. EU Member States, alongside Iceland, Liechtenstein and Norway, are also covered by the compliance cap-and-trade market of the EU ETS. Select countries also use ETS operating at a national level, including the non-EU members United Kingdom and Switzerland, not covered by the EU ETS. Data on European countries using a national ETS were obtained through the most recent World Bank Carbon Pricing report^70^.

#### Biodiversity offset markets

To deduce which European countries use biodiversity offset markets, we collated information in previous reviews^71–73^. Following the initial scoping search, scientific and grey literature was identified for each European country, using keywords and phrases: ‘ecological compensation’, ‘biodiversity offsetting’, ‘biodiversity compensation’, ‘ecological compensation market’, ‘biodiversity offset market’, ‘habitat banking’, ‘biodiversity banking’ and ‘biodiversity credits’. Relevant literature identified included: peer-reviewed scientific papers, policy documents, organizational webpages (NGO and government) and book chapters^71^.

We identified countries with biodiversity compensation policies, then identified whether there was evidence for the use of an offset market within this policy: a market-based mechanism involving sale of credits generated through restoration of biodiversity. We included markets that were operational and not those that are development (for example, Finland^74^). We noted circumstances where financial compensation is required for losses of biodiversity, but is directed towards a government or centralized body, rather than towards purchase of credits in an offset market.

### Development of interview guide

The authors began with a predetermined overarching question set by the terms of their grant agreement (EU Horizon 2020 project SUPERB (systemic solutions for upscaling of urgent ecosystem restoration for forest-related biodiversity and ecosystem services)): “what are the opportunities and barriers to the upscaling of restoration finance in Europe?”. Three workshops were organized in Cambridge (United Kingdom), Amsterdam and Copenhagen in 2022–2023 with high-level biodiversity finance stakeholders from predominantly financial institutions and other biodiversity finance-related knowledge firms and environmental NGOs with a focus on private conservation finance to gain preliminary insights and identify key questions for inclusion in the semistructured interview guide. These focus groups highlighted the critical enabling role of public policy in biodiversity finance, leading to the inclusion of a section on public policy in the interviews. The interview guide was then developed and refined following input from members of the authorship team. We developed slightly different interview guides for generalist biodiversity finance experts and investors, with the interview with investors focused on generating more detailed insights on the specific decision-making underpinning investments in conservation and their barriers (interview guides in Supplementary Note).

### Interviews

We identified relevant experts to interview through existing knowledge of influential individuals in biodiversity finance networks (acquired through coauthors’ participation in various European international biodiversity finance-related research projects, business-biodiversity fora including the Danish Nature Fund, advisory roles to the UK government regarding biodiversity net gain), the authors of relevant policy and industry reports and snowball sampling. We approached 34 experts of which 25 accepted our interview requests, spanning investors (5), directors at biodiversity finance knowledge and investment firms (5), finance team leaders or directors within conservation NGOs (5) and multilateral development institutions (4), directors of mixed nature finance-related consortia (2), a senior policy-maker (1), an academic (1), a director of a conservation brokerage/marketplace (1) and a director of a carbon project developer (1). In focus groups, participants perceived that England was the main European hotspot in biodiversity finance practice and innovation as a result of the creation of several new markets in the post-Brexit period of regulatory reform and withdrawal from the EU Common Agricultural Policy, as well as specialisms in financial innovation in the City of London. Therefore, our final sample included interviewees working in the following countries: England (9), the Netherlands (3), Denmark (2), Switzerland (1), Germany (2), France (3), EU-wide roles (4) and the United States (1). Note the explicit research question we were asking related to how to upscale investment, which makes the prior assumption that upscaling of private finance is desirable, and we therefore did not target many dissenting voices in our sample; our interviewees were therefore biased in favour of increasing private investment opportunities and many legitimate opposing views are not represented in our dataset. Interviewees were contacted via email, which included a participant information sheet providing the background and context of the study. Interviews were conducted via Microsoft Teams. Interviewees were asked for permission to record and transcribe interviews and, if permission was not granted, then notes were taken with the interviewees consent. Formal consent was secured orally from all interviewees.

### Data analysis

Interviews were transcribed using a mixture of the built-in transcription software of Microsoft Teams and a university-approved transcription services provider; all transcripts were fully pseudonymized and all identifying information or information about their organizations removed from final transcripts. We conducted thematic analysis of interview transcripts, following the sequential process of reading all transcripts (data familiarization), generation of initial codes, identifying themes, code consolidation and recoding and writing the paper alongside recoding and identifying final themes^75,76^. Most of our analysis was inductive, a bottom-up approach to coding where the researcher identifies a specific question and then identifies all codes in the data that they interpret to be of relevance to answering the question, before coding and theme identification (that is, coding is undertaken as far as possible without conforming to previous theoretical knowledge of the topic). A single subtheme was informed by our previous theoretical understanding (see section on ‘Mismatches between finance and ecology’) as this has been a key focus of the primary analyst’s research^7,11,55^, and so coding for this subtheme alone would be better described as derived from theoretical (rather than inductive) thematic analysis^76^. In line with related interview studies^19,77^, our full thematic framework describing all themes, subthemes and final set of codes is given in Supplementary Note, along with exemplar quotes evidencing each subtheme.

### Ethics

This research received ethics approval from the University of Oxford’s Medical Sciences interdivisional ethics committee (Ethics Approval Reference: R83938/RE001).

### Reporting summary

Further information on research design is available in the Nature Portfolio Reporting Summary linked to this article.

## Supplementary information

Supplementary Information Supplementary Note.

Reporting Summary

## Data Availability

Anonymized interview transcripts retained by authors in line with ethics agreement; please contact author for further details.
