# Supplementary material for: The current state, opportunities and challenges for upscaling private investment in biodiversity in Europe
Source: Nat Ecol Evol. 2025 Feb 5;9(3):515–24. doi: 10.1038/s41559-024-02632-0 (PMC11893444; doi:10.1038/s41559-024-02632-0)
Supplement: Supplementary file 1 — Supplementary Note. [file 41559_2024_2632_MOESM1_ESM.pdf]

nature ecology & evolution

Supplementary information https://doi.org/10.1038/s41559-024-02632-0

The current state, opportunities and challenges for upscaling private investment in biodiversity in Europe

In the format provided by the authors and unedited

Supporting information for "The current state, opportunities and challenges for upscaling private investment in biodiversity in Europe"

Indicative quotes in support of themes

Opportunities for upscaling private investment in nature in Europe

Theme 1: Large-scale economic and political changes opening opportunities for private investment

- *Sub-theme: Underlying political changes – lack of faith in government, and increases in societal expectations and culture*

P12: "So I think the main thing is just the recognition that we are in a nature crisis."

P7: "Yeah, for me, honestly, it's the summer of 2017 in Europe at least where it was so hot for the first time. And I I think we're used to it now, but that - all of Europe was writing about, this is climate change, which strictly speaking wasn't right. But that really started a movement in creating a market to kind of - also an explosion in what we were doing in just in terms of clients."

P2: "So we can continue to think and expect that governments will solve our problems, but they don't. They have consistently proven in the last 50 years of my existence here on earth, they don't- especially not now that that confronted with a lot of political... so you need to focus on private markets, and you need to make that work."

P4: "And what gives me hope for optimism? Or yes, umm? I think we maybe a pessimistic way of looking at it is that I don't have a lot of faith in other solutions."

- *Sub-theme: regulatory and policy drivers of demand for private investment*

P15: "What excites me is the TNFD, and the commitments in the global biodiversity framework, for example, for corporate reporting. And, if we can get some basic biodiversity metrics clear and prioritised, and everyone's sort of reporting and capturing, then countries can then force or require at least publicly traded companies to report on their impacts. The way it is now is like if you're a company and you want to do the right thing, and with your supply chain or with your water use or anything like that, there's no benefit for you to do that. The playing field is not level. There's no place you can really report that and get a tax break or anything like that, so there's no incentive to do that. Therefore, if you're doing it, you're kind of going against your profit, whereas your competitors are polluting and using externalities and benefiting from a lax regulatory environment."

P2: "…then there is reporting requirements now coming in for corporates, and there are just more and more people who want to do something."

P5: "…there's a lot of activity looking at minimising the risk, or avoiding the risk, or do no significant harm activities in the EU taxonomy. You're starting to see now, those financial instruments starting to feature as a condition component, like: I'm gonna provide this financing to you but you have to prove to me that there's no deforestation involved in any impact of the activity, for instance. Or, you look at the sustainability linked financings, where it says: I'm going to give a loan to you but if I find out that there's harm done, or you failed to achieve a certain target that you committed to me you're going to be penalised. So those are all like ESG integration or minimisation of risks kinds of activities."

P14: “So at this point of this net gain market is a big area of focus for us as it is, well, presents a revenue stream that is is alive and active today in certain parts of the country that have moved ahead of the national legislation. But, also we're sort of gearing up for that national market at the end of this year and and indeed, one of the funds that we are in the process of launching and have designed has a specific part of this net gain strategy. And so we will be looking to invest in habitat banks and hopefully habitat banks of high quality.”

- *Sub-theme: drivers of supply of projects*

P2: “Many land owners that I met said literally you know this land, when I was young come and we had this land for 6-7 generations, there were birds there were frogs, there was life, right now it's just desert, literally desert, is just sand and rocks. So land owners, they see it, they see their land has been exhausted by decades of over intense farming. Costs of farming are going up, because they need more and more irrigation, they need more and more fertiliser, while they're not getting much more for their produce. That is, on the land owner side.”

P8: “And interestingly when I'm with my our Land Management director, we've been in meetings with other land agents like looking at at sites, aerial, you know like Google Earth images of fields. And he's like, oh, look, you know, you can just tell look, you can see where all these crops have failed in this image, proximity to, you know, to a canal or watercourse. It's, you know, it's just not that, that farmland is not farmable. There's very clear patches where it's... but they're just pushing that, you know, they're just trying, like flogging a dead horse, just trying it.”

P16: “And that is where I believe the current momentum around biodiversity credits is really coming from. Because people are seeing it as an opportunity to make money. So for example, I sat in a meeting with a large European energy company and we were talking about some of the plans that they had around biodiversity credits. And effectively what they were talking about taking the experience that they have gathered over many, many years of onsite biodiversity management that they felt they were doing very, very well because they've been confronted by it, they've been challenged over it, and they've got really good practice in it as far as they're concerned, they wanted to apply that now to new projects where they would look at carbon offsets, biodiversity offsets within the same location. And I asked them the motivations around it, and they saw this as being part of their business portfolio.”

P24: “…the people who are really going to be making money from this are the ones with the most limiting factor in the whole system, which is land. And so if you own a lot of land, because you're a farmer in Southeastern England, and the government is pushing to rip up the Green Belt and put houses everywhere, and at the same time introducing a biodiversity net gain requirement, then rewilding your land and transforming it into the relevant kind of habitat might be more profitable than farming it.”

Theme 2: Opportunities arising from innovation and experience

- *Sub-theme: technological innovation as an enabler*

P11: “I do think technology can really help with because you have the transparency, Yeah, you can get a satellite image of a place very easily you know, to see what's really going on and so, That that will, you know, quickly catch out anyone who's really, really trying to game the system.”

P6: "…in these markets that the increasing use of technology as part of the monitoring process that helps in tracking the actual impact using technology. There has been I believe some attempts in the use of blockchain but I don't know where they are, I admit I'm not an expert on that".

P2: "The outcomes for nature might, if you measure by diversity for example with eDNA- so eDNA measures the number of species but also the variety of species, so they measure how many animals there are of a certain species, but also how many species even over time, it can measure the variety within a certain species which is ultimately even more important than the number of species. But anyway that is like a hard given number, you can measure that. And you know even for like some large tree planting projects you hardly see an increase in biodiversity because all the trees are the same age, same type, even the same DNA often because they're all the same like from the same seedling… so that is again something you can measure. Again on carbon you can measure I mean, there is so much technology right now, even again with cameras come up with drones, that you can take, that you can measure- even soil carbon, even from satellite."

- *Sub-theme: the development of interdisciplinary experience and financial innovation through learning through time*

P9: "I think at the moment the basic carbon credit model is the most interesting and, and that's and that's because they're recognise – there already are recognized standards. The buyers of those credits understand the market they've, they've learned over decades of how of how of like what they should be buying, how to assess integrity, what the, you know, you know, what the due diligence process should be like."

P18: "Then you need, like, what we have is this [nature fund], it's aggregation models. So a lot of these projects, especially across Europe, are going to be like the UK - really small and they need to be aggregated up to reach a size..."

P14: "So the way we're looking to deploy capital in [our nature fund] is to encourage aggregation approaches. So that's where typically you'd have the same developer performing the same sort of activities for restoration for a given revenue stream or given market, nature-based solution market across a number of different sites, perhaps different land owners, but operating with this broadly the same transaction structure, so types of contracts, umm, uh, broad contract terms and and being able to aggregate those up into sort of a vehicle or one single transaction. So that that's the way we're looking to try and get to smaller projects. And that's a sort of a helpful approach because it basically means you can standardize your due diligence approach as an investor. And there's still sort of project specific or site specific DD you'd have to do. But you can optimize that process quite substantially if you got the same developer and the same set of contracts."

P2: "So we have structured that in a way where land owners one they make a permanent commitment, two there is a guarantee payback, that is normally five years, so any money that went to the landowner if nature is destroyed for reasons out of his control- if it's fire you cannot- but if it's for a reason that's in his control, like mowing it away or whatever, he needs to repay it."

P14: "We'll have another layer of what we're calling developer managers that are potentially land agents, or other ecological specialists, the likes [land agents] and all those kind of people who will actually review the developers and making sure that they are meeting their milestones for delivery, especially during that that restoration period and and they will be sort of approving those sort of payments, they're gonna go to those developer partners for for delivery of those services. So and we'll be sort of, you know there's there's ways that you can for example withhold back some of those

payments to developers until they've they've got that independent validation of the site being delivered.”

P10: “So I would say the big reason is the number of skilled financial intermediaries and people who can, yeah, basically intermediate between nature enterprise models related to nature and mainstream finance and impact finance.”

Challenges to upscaling investment and its ecological and social benefit

Theme 1: lack of high-value revenue streams

P17: “And I would consider us very risk-tolerant investors, very, in the grand scheme of things. And we're looking at things that others would definitely not look at. But even for us, so many of the projects we see are just... It's not clear where the revenues are going to come from. If they do have a sense that they have to assume crazy prices to make something work on a kind of risk-return basis. And it would be much better to say, actually the prices are going to be very sensible, but it's also very low risk because we are part of this governance scheme that's going to guarantee us some kind of floor price, whatever, it would really, really help.”

P18: “I sort of see as you've got this long-term arc, which is reporting being mandatory, companies understanding they need to reduce their impact. You've got taxonomies that are working to support them in that. And then they're accessing green finance to help them do that. And then you end up maybe like by 2050 where companies have significantly reduced their impacts and we're in a better place. The problem is that doesn't really pay for restoration.”

P20: “So yeah, so so the the basic evidence of reliable cash flow or something you can secure to rise against, for example, is is is shaky.”

P15: “I think that they're [large private investment funds] recognising how challenging it is to actually find investible projects, so pipeline is a big barrier there.”

P14: “I mean, I wouldn't look at it as a cut because yeah, I mean on the net, but even though the projects need to hit that threshold, you know, if if X percent of them fail on a net return, our fund will probably actually return about, you know, 4 or 5%...And and that is pretty low. So especially when you can get 3% by just putting it in a government bond. So yeah, it's that's the way it this way it is.”

Theme 2: risk

- *Sub-theme: political and regulatory risk*

P4: “And I think with biodiversity credits, I think it's - and any type of credit – it’s gonna be difficult to make it work. And I think there's a lot of challenges, and and the biggest challenge will be political will, because I think the success of these markets hinge in the political will.”

P20: “And it's it's just an interesting tendency by by donors and and and think tanks. Uh, and [they] used to sometimes bring the most complicated financial cookery wizardry to the least bankable sectors of the world. And and and I saw this in the water sector, where [various multilateral development banks], everyone really wanted to because it was kind of revenue generating. They

they try to to pull out financial engineering into it. Uh, whereas that any moment Treasury could just go out and just issue another bond, in both cases, you know for the immediate needs."

P19: "Well, very easily. I mean, I would say the lack of political ambition translate across all instruments. The same lack of political ambition to put in place regulation has translated, for example, in an EU ETS where we issued far more credits than actual emissions for more than a decade. You cannot address lack of political ambitions through different tools, and you cannot expect private business who has, by definition, a short-term horizon, and is focused on making money, and so be it, so it should, to care about the public interest."

P9: "…in terms of making finance available, to me, the worst thing [government] can do is increase uncertainty about what's gonna be, what's gonna be included or excluded under a a new regulation scheme. Because I think that certainly if you're looking towards more pure commercial finance, which I'm not sure, you know, most of the money that we're talking probably to is is, yeah, some of it is pure commercial, but some of it is more kind of soft commercial or or starting to be kind of mixed commercial and philanthropic. Yeah, I think any increased uncertainty will reduce appetite to to to make big investments in this area."

P18: "Yeah, so nutrient neutrality I know there's been like a five million trade done out of environmental farmers group on that already. It's a little iffy, nutrition neutrality we don't even know like how rigorous it is and how long it's going to be around for. So I'm not sure it's something to really jump up and down about."

P17: "But from an investment climate perspective, it remains one of the markets where investors are putting money into improved forest management or direct restoration, partly because they're sure that that's going to keep going or they seem certain it's going to keep going, and the rules if they change, will change. And that's fine. You can just kind of be strengthened over time. So in a funny way, I think the magic of policy is you don't have to get it right the first time. You can kind of continuously evolve and get better and as long as the direction of travel is clear, but you're not going to go backwards, that creates the right conditions for investment."

- *Sub-theme: mismatches between finance and ecology*

P24: "…there's an amazing number of people who are putting their brain power to developing these metrics and stuff. They're hoping to make a living out of it. Most of them will not. But that's how these things, I guess, grow and evolve. And they will consolidate into a few preferred methodologies that will meet the needs of the corporate buyers."

P24: "No detailed metrics. Non-mandated by law. But quite a few people have tried to come up with methodologies, and some of them are being successful, generally the ones that are quick and easy. So area of habitat with a multiplier, very basic stuff."

P1: "Ja, that a good question that I unfortunately cannot answer as I haven't interacted with so much with my [investor] colleagues who are [investing in] organic agriculture in the EU. I suspect that they rely on - so, from – that they rely on certification."

P17: "BNG for example, we think we're at the point probably where if... And there are a couple of developers that are creating BNG units. We haven't actually run the numbers on the investment returns, but assuming those work, that would be investment ready, because there's efficient momentum in that market. It's clear enough. Of course, there's flaws with it, as you've pointed out in some of your research. But that's kind of besides the point. From an investor perspective, that will be worked out through academic work and policy work."

P9: "…we're seeing increasing numbers of financial institutions who are only willing to put in upfront project finance or large scale investment to already existing standards, and so therefore you are being required to adhere to VCs or gold or uh, you know, whatever other standards they might, they might recognize."

P9: "That's your worst nightmare that you end up named in a Guardian article for what you're doing or what you've contributed to isn't having the impact you said it is."

P4: "So I mean, there's also the role of NGOs, right, and journalists and I think so, one, one way you can sort of steer private actors behavior is through loss. But the other one is through through the threat of reputational damage. And I think that could also be or obviously that can also be a crazy big motivator to do good. And I think that, I mean that we've seen also with like, deforestation commitments right. That I mean, these cocoa companies there are terrified of these reports coming out where they are, could be blacklisted for, for deforesting, and they may not even know that they are right or that they are working with farmers who are deforesting."

P11: "It's just literally [farmers] need a big chunk of change up front to cover the opportunity cost of saying no to their current revenue generation, and it's like who covers that income that that opportunity cost or and actual costs of of, of of withdrawing from the current funding mechanism?"

P10: "You have for example legal and regulatory bottlenecks to bringing private finance in. So if we think about communal conservancies, CBNRM community forestry, they have potential business models. The problem is they don't have legal tenure on the land and they have a governance model, which is not familiar to you know, the mainstream finance community at all. So like, if you're like my whole family wants to take a loan, the bank will be like, yeah, but who's the primary applicant, And you know, who could we actually chase for that, like, your whole family can't apply, but..."

- *Sub-theme: risks from social perceptions and inequities*

P7: "We are lucky to be honest. In [location], we work on private land also very - how do you call that - thinly populated, lightly populated areas? Same goes for [location], very lightly populated and same goes for [location]. So we're a little bit lucky in the social, you know, in the in terms of putting a forest where people don't want forest for instance, we don't have that a lot. We did have that in Africa. So we've learned from that. It still is very difficult, challenging. Not everybody's always is going to be happy with with putting a forest somewhere.

P4: "And I mean, we can think of this as like, like the moral thing of it, right. But also, if people are not benefited from a project, they're just gonna keep deforesting right, I mean as would I if I needed to, like, grow food. So absent, I guess, military violence or absent, like, super strong ways of keeping them off the land, the restoration project or the conservation project is just not gonna sustain."

P8: "I'd love to monitor, but we've got, we do actually have a a big program planned for ecological monitoring because we've got that in house, we've got that expertise. So there's loads of species that will be monitoring, we'll be doing soil, soil carbon, soil structure, deep wells, measuring water. So there's a lot of other stuff that we're doing, but because we don't have that social expertise in house, at the moment we're not planning anything more than occasionally check, you know, checking out assumptions of [an ecosystem services decision support tool], whether that that sounds a bit shit, but that's just life.

P10: "So I I think it sounds a little bit depressing what I'm saying, but I just feel like it needs public finance to take one more step and then, you know, I think these pooled vehicles would be, you know, a good option, but they're very hard for citizens to understand. And most, you know, public finance

ultimately needs to be accountable to voters. And if it seems like it's going into like, JP Morgan's account, what voter is going to be like, great, let's give our nature money to JP Morgan or whatever. So yeah, it's it's a bit tricky. I think at this point without you know more public de risking, and also like I think there's a communications piece to help people understand, You know, I often I think about for example like what would happen if the Daily Mail picks this up? You know, it's like the Daily Mail would be like ohh, you know, whatever, Commonwealth Development Corporation gives Citibank 5,000,000 or you know it just it looks bad."

The role of public policy

Theme 1: upscaling private finance no substitute for regulation

P15: "And then the question is, well, why don't nature-based businesses have 20%, 30% internal rates of return? And a lot of that comes back to regulations in that the asset values are not correctly attributed, water is too cheap. Very high quality wood is being over-exploited right now, so the prices are too low. If you did it all sustainably globally, if you had a moratorium on old growth forest or something like that, the price of the wood would be higher."

P17: "I think without a legal framework to stop the destruction of intact or relatively intact ecosystems, no amount of investment is really going to change that, I think. And so we already have a framework of rules that's helpful, but strengthening that, it shocks me that we still have native forest conversion in Europe today."

P17: "So there's a set of things that should just be banned in my view, and that will really help because it'll help investors. It will drive up demand for, for example, products that can revitalize highly degraded soil. Why is there less demand for that? Because there is still an option of additional conversion across the world."

P4: "I always thought, well, I mean, there's such a big market for sustainable project products and so on and sustainable chocolate and everything like that. But it's still a massive market for it, unsustainable cocoa, for example. So you're always gonna win taking the current economic system. We always gonna win by not being Sustainable and I think if you have strong regulations that can level the playing field."

P11: "So states, 'cause they set the rules of the game, they can encourage all require the avoidance or reducing impact which is the first part of reducing biodiversity loss. So that is what they should be doing."

P15: "…we do a lot of work with protected area finance. There's a whole range of specific mechanisms that are promising there. The data on general biodiversity funding suggests that governments are the main source of funding for nature. They should be. These are public goods, they're shared resources, they're complex ownership, and different beneficiaries, and so government budgets are really important. Results-based budgeting being one of the great opportunities there to enhance and increase the effectiveness as well as the amount of money that's going to nature if you can explain why you need the money, which has been a barrier. I'm sure we'll get to that in the future."

Theme 2: Public policy as the great enabler of biodiversity finance and its own worst enemy

- *Sub-theme: tensions between getting land and preventing access to land*

P20: "… whereas a mere transaction of the ownership of land in in the minds of many economists, isn't the thing now. Personally, I disagree with this, but I'm just explaining some of the underlying economic thinking here, and it's linked to this whole additionality question also of conservation versus, you know, restoration. You know, there's a lot of impact investors that struggle with conservation for similar reasons. You know what? I just buy it and I sit on it. They they can't quite see themselves in in that, but but the the other side of the coin is that unless you actually address that question, you might as well go home because it's all about space and land."

P20: "I mean the harm, the harmful subsidies play a massive role here. I mean, joy, it's a completely uneven playing field. You give me free money to the competition here. You know that that that is a major issue and it it also inflates so much of the, the the land that you would target for biodiversity is precisely the kind of land that is often only viable commercially because it's getting subsidies. And so it pushes up the value of that land. The opportunity cost of doing something on that land and so it has, it has a almost a multiplier effect of bad uh, destructive issues. So, so and and and given that about half of Europe space is agriculture, that at one level or another is within some kind of a support framework. Umm that that is a big issue. Uh, so, so conservation organizations have to spend so much of their budgets on the net, present value of future harmful subsidies, basically, to buy people out. It's nuts."

P9: "One of the issues we face as an organization in in every enterprise element that we do - is we have to - our starting point is that we have to find a way to compete with the agricultural subsidies that people can access, and this is a big, a big problem for us."

P9: "…looking at a quick financial assessment like OK, if they're currently getting subsidies and the farmer says I'll only do this if you match what I get, the profit that I make at the moment from my activities then that gives you an idea of what you have to have to match it financially and then that starts helping you develop a kind of financial model."

P24: "A second source that we're trying to pursue is, okay, how can we use subsidies in support of rewilding? We see that as a major revenue stream that is keeping some of the non-productive farming still ongoing in Europe."

P3: "And one one more, the like critical factor is the price. Because we have, uh, until now, said to ourselves that we don't want to spoil the market. So what we do is that we we we we use, Uh, you know real estate - people working normally in the market with buying and selling of farms and forests and everything, and they do, they they the judgment for us, how much is this worth? How much should we pay for it in the market? What is the market price? And of course, we can negotiate around that place, but we keep to that price. Uh, we we could do more if we just said let's put 30% on top of the market price then would be more, much more easy to to to to make projects of course. But until now, we we stick to the market price plus minus something. But not much. 10% maybe."

- *Sub-theme: saving private finance from risks the state makes itself*

P20: "I mean when when you, when you then look at things like the voluntary carbon markets, the questions from the credit risk teams are the same, I mean and and it's really has to do with the fact that it, the you know it's, the the weakness of the regulation around it, really.

P7: "Yeah, legislation is an obvious one. So in Spain, for instance, we do a lot of our contracts are for 40 years and all of a sudden last year, for instance, government said, hey, look, we only are in power for four years. So we're not allowed to or we don't feel comfortable in signing these convenants as

they're called for 40 years. So that gave a lot of stress in the market that has been solved by the way. But those kinds of, you know, changes in governance or in policy are really difficult."

P1: "Yeah, it's called blended finance, and it is that – and I'm not the only person working on this in the financial sector. There was initially a lot of excitement in finance – I think now reality has [inaudible]. Blended finance means combining different sources of capital in a way that allows you to achieve high impact goals, while at the same time achieving scale. And that is generally done by taking public sector money or philanthropic money to for example go in as first-loss tranche, and then it is much more easy for more commercial and eventually also retail money to go on top of that, because the public sector or foundation or whoever it is, would sort of take the first hit."

P14: "I think well, first of all, blended blended capital I think it's an important one and that's the basis of our [nature fund]. So that's that's essentially stacking public and philanthropic capital that's perhaps concessionary or structured as as first loss. So it takes a first hit if underlying investments fail. structuring that in the capital stack together with, to attract more mainstream capital in and that can help solve for the lack of investment capital available so that's the the structure of the [nature fund]. So essentially the the, the larger institutions come in sit behind that, that first loss capital and it acts for the downside buffer for them, that's a tried and tested mechanism that operates in nature based solutions funds around the world and and social impacts and and renewal energy funds."

- *Sub-theme: coordination*

P10: "You know, there basically needs to be an assessment where it's like, OK, we see viable business models in these types of nature for these types of reasons. But we're still gonna need public finance and regulation and sticks or exclusions or whatever it is for these needs. And we're nowhere near that kind of level of assessment. Now we're just at the, you know, front of it, scratching and saying, like, maybe this might work, maybe that might work. Here's a few little transactions that have been interesting, but you know, they haven't been proven or scaled."

P20: "So in our case, our main competition, as it were, what were grants, and and the lesson from that is not, you know grants are are bad. No, I think our conclusion was no, grants are definitely necessary. It's just that we need some kind of a much better coordination of the two, because if you have a a debt instrument like ours, it goes into the market, then you know you trying to develop a case any minute, they can jump ship and and and go with a grant or, say mobile concession finance or whatever. So that that kind of muddies the picture a bit in some ways. Uh, in in Ireland, you know, we we started looking at peatlands, what happened halfway into the discussions, the [a grant-making body], you know, BAM, 100 million. You know you can't compete with that.

- *Sub-theme: distinctions between public and private finance are arbitrary*

P15: "And everyone's so excited about private sector money and private sector investment, stuff like that, but people just don't realise how important government is in creating conditions for private investment in nature. And it's like, "No, it's got to be the private sector," it's like, "What is the private sector? What does that mean? What's a private company?" "Oh, well, it's a company that's registered." "Registered to who?" "Well, with government." "Why? What does that mean?" "Oh, well, it has to follow the laws. It requires the courts." There's no such thing as private sector versus public sector. Everything's integrated, everything's blended, and we need that."

P16: "That is one that I think has to be, you've got to instigate that at a system level. I don't think again, it's one that's going to happen organically within our economy on its own, our economy, every economic actor plays according to the existing rules of the game. And yes, some innovation happens

that changes that and pushes people on and makes things move. But ultimately they're constrained by the operating environment within which they are, within which they exist. Everyone's constrained by their reality. And I think the only force that can fundamentally shift that reality is through the role of the state when it comes to things like this and the timeframe that we're talking about.”

P19: “I mean, someone like [an industry commentator] has also written extensively on the fact that he believes that regulation is the only thing. I mean, obviously different governments have different priorities, but I would argue that the state is the only entity with the mandate, and democratic legitimacy, and possibility to care about the public interest. That's why I strongly reject the Davos narrative that all business must take the lead, blah, blah, blah. It's a question of underfunding. And private sector has a key role to play, et cetera. Because here, again, you have a lot that is unsaid. Yes, private sector has a key role to play in obeying regulation. That is not a key role. It's just to obey the law.”

P6: “And then you move into the next phase which is how do you integrate that into the core of your operation which is not yet happened and I think we're moving into that phase. And that the role of the regulations and the policy becomes quite critical because I mean you just need that nudge.”

Full thematic framework

**Theme, sub-themes**	**Description**
Challenges	
enabling damaging values	concerns about markets enabling values that could conflict with the goals of conservation
lack of supply	lack of supply of projects to enable private investment
for high integrity	lack of supply for high-integrity projects
land	lack of land availability
land tenure	how land tenure affects environmental outcomes and market participation
Mismatches between needs of investors	inherent tensions between the characteristics of project financing/investment and ecology
biodiversity is where poor people and institutions are	spatial overlap between areas of high biodiversity and poverty and weak institutions
challenges of commodification	challenges of environmental commodification
carbon biodiversity trade-off	trade-offs between optimising for carbon revenues and biodiversity outcomes
non-fungibility	inherent complexity in nature renders it non-fungible
uncommodifiable biodiversity	some forms of biodiversity (e.g. conservation that does not deliver a measurable uplift) are challenging to commodify
lack of high value low risk revenue streams	lack of revenues streams that have risk-return rations high enough to attract mainstream investors
Measurement	the measurement of biodiversity
additionality	the additionality of conservation investments
easy weak KPIs metrics	the use of key performance indicators which are easy to measure but correlate poorly with real world conservation outcomes
temporal mismatch	mismatches between the relatively short time-scales over which investors need returns and the long time-scales of conservation
speed for markets to mature	new markets time a long time to mature
upfront capital intensive	the need for upfront capital for conservation but delivering long-term gains
uncertainty and risk	various forms of uncertainty and risk facing investments in nature
ecological uncertainty	inherent ecological uncertainty from features such as natural stochasticity
public opinion	risks from public opinion and attitudes
Greenwash	marketing of environmental benefits that are tokenistic or false
regulatory uncertainty	uncertainty in whether the regulations will remain unchanged, or be repealed
reliance on standards	relying on certification or the achievement of some kind of standard as a proxy for a positive conservation outcome

Theme, sub-themes	Description
non-compliance	problems with projects not complying with standards
reliance on good-will	systems rely on good will to go beyond the bare minimum acceptable under the standard which may have limited benefit
sensitivity to financial risk	investments susceptible to various forms of financial risk
unproven track record	conservation investments do not have a long proven track record
historically poor outcomes	conservation investments have historically underperformed relative to their stated ecological goals
unwilling to pay price of true cost	investors unwilling to cover the full costs of doing effective conservation
transaction costs and complexity	transactions costs involved in the governance of a conservation investment
politics	politics and political wil influencing nature investment opportunities
resistance from finance	lack of willingness from financial actors to shoulder the institutional work of making nature finance work effectively
social injustice	various forms of perceived social injustice around nature investments
concern the wealthy benefit	concern about how wealthier population groups stand to access and benefit more from nature markets
international injustice	international inequities in nature markets
lack of local engagement and beneficiaries	issues relating to insufficient engagement with local communities or those affected by projects
safeguards	safeguards put in place to mitigate social risks or impacts
Opportunities	opportunities for upscaling private investment in nature
better than the alternative	nature markets perceived as better than the alternative scenario
challenges facing farming	economic and cultural challenges facing farming increasing opportunities for nature markets
creation of new markets	revenue streams created through establishment of new markets
Demand	changes in the demand for private investment in nature
CSR	corporate social responsibility
ESG dependencies supply chains	addressing supply chain risks or increasing ESG scores through mitigating risks
nature-positive	corporate nature-positive commitments
Net zero	corporate decarbonisation commitments
ordinary consumers public opinion	changes in public opinion demanding nature loss is addressed
Policy drivers	policies perceived to potentially drive increased private investment
Suppliers new opportunities	new economic opportunities for land managers
Voluntary frameworks	sector-led voluntary potential drivers of increased investment

Theme, sub-themes	Description
precursor to regulation	voluntary initiatives as a precursor to adoption in regulation
Financial innovation	opportunities arising from financial innovation
Aggregation	aggregating multiple smaller projects together into a larger investment vehicle
new ways of ensuring outcomes contract design	novel mechanisms for tracking the ecological performance of investments and embedding these in the design of contracts
More expertise	increasing professional expertise in nature finance
experience and practice	learning by doing
technological progress	technological advances creating new opportunities for investment
public policy	the role of public policy in influencing private investment in nature
better management of public land	improved ecological management of publicly-owned land
Coordination	coordination issues affecting private investment
Coordination between funding streams	coordinating between different sources of funding for investment in nature
Coordination between stakeholders	coordination between different institutions to deliver funding outcomes
creation of new markets	governments creating new nature markets to generate revenue streams
enabling conditions	enabling conditions for upscaling private investment
Knowledge sharing	increased knowledge sharing within the nature finance community
mandatory disclosure	making the TNFD mandatory as a driver of investment
property rights	addressing property rights issues to enable private investment
public data	making key datasets public and transparent
standardisation	standardisation in processes and certification
Regulation	regulation to upscale private investment
better enforcement	improved enforcement of existing regulations and ensuring compliance of nature markets
claims	regulating claims derived from purchasing nature-based credits
direct regulation of harms	increasing the stringency of regulation preventing biodiversity loss
financial regulation	financial regulation affecting investment in nature
poor accreditation methods	improving the quality of accreditation methods
regulation holding back nature markets	perverse regulations which act as a barrier on upscaling private investment
Subsidies public investment	subsidies and direct public investment in nature
Barriers to buying public land	barriers and rules preventing the acquisition of land for conservation
Blended finance	project financing where governments or philanthropic donors underwrite some of the risks for private investors

**Theme, sub-themes**	**Description**
projects on the margin	changes of risk:return ratios tipping projects over the profitability margin
Risks	risks associated with blended finance
investment in public goods	direct public investment in public goods
subsidies competing with markets	competition between subsidies and the revenues from participation in nature markets
taxation	tax issues affecting private investment in nature
Skills and capacity	skills and capacity issues in the public sector
low risk appetite	low risk appetite of the public sector

Public policy to enable upscaling of private investment opportunities in Europe

Policy proposal	Participants
Better management of public land	
Improve management of public land for conservation	7, 14
Changes to tendering contract durations on municipality owned land to enable private-sector acquisition of contracts for long-term land management	9
Changing spending rules relating to land purchasing	
Enable public money to be spent on purchasing land for conservation	20
Policy coordination	
Landscape-scale planning to ensure disparate conservation projects receiving various forms of investment add up to regional and national conservation goals	3, 12, 15, 24
Promoting connectivity between biodiversity protected on private lands and increased consideration in conservation spatial planning	16, 24, 25
Coordination between difference sources of funding (i.e. delineating the role of private and public finance)	9, 10, 14, 15, 16, 20, 21, 24, 25
Coordinating between policies and government departments (e.g. agricultural subsidies and nature markets)	8, 13, 15
Clarifying accounting rules for carbon credits across jurisdictions (i.e. preventing double counting, fix disparities in tax such as VAT)	2, 7
De-risking	
De-risking through guarantees, providing government-backed floor prices on nature-based credits so that farmers can enrol, insured against market collapse	1, 8, 10, 14, 15, 17, 18
De-risking by providing up front support for projects when risks to private investment are highest	10, 11, 12
Repurpose public development banks so they can seek concessional rather than market-rate returns	12
Subsidy reform	
Providing subsidies for the ongoing management of conservation outcomes, rather than just up-front investment	14
R&D support for nature technology companies	17
Subsidising sellers of environmental commodities (e.g. by part-matching the price of private purchases)	2, 4
Greening agricultural subsidies	2, 9, 11, 13, 24
Scaling up environmental markets	
Bringing in further biodiversity-related compliance markets	4, 9, 14
Mandating that a given percentage of carbon credits used in emissions trading schemes need to be invested in natural climate solutions	14
Increase the level of compensation required in compliance markets above no net loss and towards nature recovery	4
Improving the integrity of environmental markets	
Ensuring that carbon and biodiversity credits achieve a regulator-backed high quality standard using scientifically-credible metrics	6, 7, 11, 12
Ensure that standards and legal liability is located in the country that has the highest regulatory and legislative standards, to align standards internationally with the highest standards	5
Networking and knowledge sharing	
Improve opportunities for projects that have received private investment to share knowledge with aspiring projects	4, 6, 18, 20, 21
Investment readiness funds which help develop capacity for project to then enter environmental markets	14, 18
Financial regulation	

Mandating nature-related disclosure	18
Biodiversity border-adjustment mechanisms	11
Provide more certainty regarding definitions and criteria in sustainable taxonomies	6
Allow institutional investors to take more risk to incentivise them to invest in nature-based solutions	1
Property rights	
Create forms of property rights that enable the protection of nature for a given time period	7
Tax reform	
Tax incentives or removing disincentives to enrolling private land into conservation	12, 14, 15
Public data	
Subsidy for conducting baseline assessments of the quality of nature on private land	18
High-quality national registries for carbon and biodiversity credits	2, 8
Make national forest and biodiversity data public	7, 12
Develop map of ecosystems around the world using the IUCN red list of ecosystems criteria	12
Monitoring and enforcement	
Clarify who is responsible for monitoring on nature-based credit projects and increase accountability	8
Improved state enforcement of land management activities delivered to generate credits for markets	4, 5, 8, 14, 15, 18
Standardization	
Standardise carbon accounting methodologies	6, 7
Increase regulatory oversight over standard setting or certification organisations	6
Regulation of claims	
Increased regulation of the claims made and reporting when purchasing carbon credits	9
Direct regulation of ecological harms	
Banning ecological harms such as damaging products, uncertified products, and habitat clearance	4, 15, 11, 12, 17
Regulation of utilities	
Relax restrictive regulations that bias utilities towards investing in grey infrastructure and capital-intensive projects over green infrastructure with lower capex but relatively higher opex	14
Public investment in biodiversity	
Increase direct public investment in biodiversity	15, 18
Upskilling public sector	
Increased investment in and training of public sector employees	1, 8, 11, 15,

Semi-structured interview guides

<u>Brokers and knowledge services firms focusing on biodiversity finance</u>

1) **Participant information sheet**
2) **Verbal consent**
3) **Introduction**
    a. *If I am unfamiliar with the participants' work.* Could you tell me a bit about the biodiversity finance work you do on a day-to-day basis?
    b. What kinds of financial instruments for investing in nature restoration are you or your firm working on?
        i. What initiatives and funding mechanisms are happening in Europe?
        ii. Who are the major or potential buyers?
    c. Are there certain funding mechanisms you believe are the most promising for upscaling restoration?
        i. *If they name a specific project.* Can you give any examples of where that/those mechanism(s) has been successful?
        ii. *How do we know if was successful?*
        iii. *Why was it successful?*
4) **The role of the state in upscaling finance for nature restoration**
    a. What are your views on what the role of state institutions should be in driving nature conservation and restoration?
    b. How can national governments or the EU help in increasing financing for nature restoration?
        i. what specific pieces or types of legislation are missing to assist with upscaling, thinking about both carrots (i.e. incentives) and sticks (stricter regulation)?
5) **Governance and design of financial instruments**
    a. *So for [insert financial instrument the participants said they are working on here]:* how do you find appropriate projects for your project portfolio?
    b. What are the environmental outcomes that the project is tied to, and how are they measured?
    c. Are the project's social outcomes measured, and if so, how?
    d. How is/will the project's environmental impacts be monitored over time?
    e. What procedures are in place to ensure the project achieves additional outcomes?
    f. In your view are there any risks that they don't deliver what they say they will?
    g. What happens in the event of non-compliance or failure to deliver the outcomes on behalf of the funded project?
    h. What kinds of enforcement mechanisms are in place?
    i. How would project funders be kept informed about the economic, social, climate, and ecological outcomes of the project, and what is your experience of how interested finders are in the details of the project?
6) **General reflections**
    a. The environmental movement has been attempting to create markets for non-market ecological goods and service for 50 years. What makes it different today?
    b. Are there any other questions that you think I've missed that would be interesting to talk about?

<u>Investors in biodiversity financial instruments</u>

1) **Participant information sheet**
2) **Verbal consent**
3) **Introduction**
   a. *If I am unfamiliar with the participants' work.* Could you tell me a bit about your organization and the role you have?
   b. What kinds of financial instruments for investing in nature restoration are you or your firm purchasing?
   c. In what regions are your projects located?
      i. Why have you chosen to focus on these regions?
      ii. Have you invested in any projects in Europe?
   d. Are there certain funding mechanisms do you believe are the most promising for upscaling restoration?
   e. Can you give an example of where that/those mechanism(s) has been successful?
      i. *If they name a specific project.* Can you give any examples of where that/those mechanism(s) has been successful?
      ii. How do we know if was successful?
      iii. Why was it successful?
4) **The role of the state in upscaling finance for nature restoration**
   a. What are your views on what the role of state institutions should be in driving nature conservation and restoration?
   b. How can national governments or the EU help in increasing financing for nature restoration?
      i. what specific pieces or types of legislation are missing to assist with upscaling, thinking about both carrots (i.e. incentives) and sticks (stricter regulation)?
5) **Governance and design of financial instruments**
   a. What are the characteristics of financial products delivering restoration that you would consider investing in?
   b. What kind of due diligence would you undertake before investing in a biodiversity product?
   c. *Based on earlier questions – find out if they invest in projects directly or invest in brokers who invest for them:* What characteristics do you look for in a project/ in a broker who aggregates and markets biodiversity investments?
   d. How do you track the social/environmental/climate impacts of your restoration financing?
   e. *If they mention having purchased a biodiversity-related financial product:* What are the environmental outcomes this product is promising to deliver? How does it work?
   f. What mechanisms are in place if the restoration project falls short of its goals?
6) **General reflections**
   a. The environmental movement has been attempting to create markets for non-market ecological goods and service for 50 years. What makes it different today?
   b. Are there any other questions that you think I've missed that would be interesting to talk about?

Details of interview participants

participant number	Description	Country
1	Director at sustainable finance institution	Netherlands
2	Managing director at biodiversity brokerage firm	Netherlands
3	Managing director of a philanthropic nature investment fund	Denmark
4	Biodiversity finance academic	Switzerland
5	Director at biodiversity finance advisory firm	UK
6	Director at biodiversity finance advisory firm	UK
7	Director at carbon offset firm	Netherlands
8	Director at nature conservation charity	UK
9	Head of finance at nature conservation charity	EU (unspecified)
10	Nature team leader multilateral development institution	Denmark
11	Director at nature- business advisory firm	UK
12	Director at nature- business advisory firm	UK
13	Nature team lead at European policy institution	EU (unspecified)
14	Director at biodiversity finance advisory firm	UK
15	Director at business- conservation NGO forum	US
16	Nature team lead at international conservation NGO	UK
17	Leader of nature investment team at asset manager	UK
18	Director at sustainable finance institution	UK
19	Director at sustainable finance NGO	France
20	Nature team leader at multilateral development institution	EU (unspecified)
21	Nature team leader at multilateral development institution	EU (unspecified)
22	Communications director at nature-based solutions investment fund	Germany
23	Director at nature-based solutions investment fund	Germany
24	Director at nature conservation charity	France
25	Director at nature conservation charity	EU (unspecified)
